# Practical Observations and Suggestions in Medicine

**Published:** 1847-01-01

**Authors:** 


### Practical Observations and Suggestions in Medicine. *Second Series.* By *Marshall Hall,* M.D. F.R.S.L.&E., Foreign Associate of the Royal Academy of Medicine of Paris, &c. &c. Small 8vo, pp. 360. Churchill: London, 1846.

At the close of our notice of the First Series of these Observations, &c., in the number of this Journal for April 1845, we expressed a hope that Dr. Hall would revise and more carefully prepare any subsequent series, so as to render it more worthy of his acknowledged reputation. The review, which we propose to take of the present volume, will enable our readers to form their own judgment pretty fairly whether Dr. H. has acted upon this friendly suggestion or not.

The Preface is amusing in its way. "The hydra, quackery, now so prevalent both in and out of the profession," comes in for two or three stout blows from our ever-vigilant author. All sound practical medicine, it is more than once declared, must be based on an accurate knowledge of experimental and clinical physiology. "The biblio-physiology of the day can issue in no good whatever. The medical mind wants discipline: we should study the works of Harvey and M. Louis; experiment, and observation, and philosophy should go hand-in-hand." A physiologist could not, if he would, either be a quack or disposed to quackery. So, at least, says Dr. Hall: we hope it may be so. "There is nothing in Mesmerism, Hydropathy, Homœopathy, that could lay hold of his tutored, chastened, philosophic mind. To the hideous folly of Mesmerism, and the disguised nothingness of Homœopathy, he would feel an ineffable disgust; of Hydropathy he would think as of any other system of gambling—with the addition that, in this case, safety, health, and life are the stakes." P. vi.

Let us not forget that too much may be made even of Physiology, as of other less important introductory and auxiliary sciences, by him whose grand end and aim ought to be the alleviation of suffering and the cure of disease. It is possible that so much attention may be paid by the builder to the groundwork, that the superstructure—the real building—is all but neglected; and that the farmer may be so much occupied in experimenting with new manures and so-forth, that he forgets in the autumn to compare his crops with those of his less curious neighbour.

Dr. Hall, with the most laudable desire to find occupation for others, suggests a number of subjects or themes for future investigation. Most of them are, as might be expected, more ingenious than very practicable; or, when practicable, very practical. For instance, "the influence and agency of mind, nerve, blood and muscle, respectively, in the animal economy"; "the reciprocity between the ingesta and egesta in the animal and vegetable kingdoms," &c. &c.;—are no easy topics, we should think. "I had once the idea," says he, "of making a special study of the physiology of death, &c., so as to give evidence in trials involving the questions of suicide, homicide, &c., my object being to succour the innocent, and not to spare the guilty. I would *suggest* and recommend this project to some young and generous-hearted physician, as not unworthy of a life of special devotion and research. But I fear there is little *poetry* in our profession, and that 'the age of chivalry is gone.'" Who the true knight-errants are, we must leave the readers to guess.

After the Preface, come the Introductory Observations. We are again favoured with some remarks upon the empiricism that is so rampant, under different forms, in the present day. There is unfortunately but too much truth in the sarcastic remark, that "there is no folly too foolish for some of those, who are the *judges* of our qualifications for the practice of our profession."

Now what is the real remedy against quackery? KNOWLEDGE (the capitals are not our's), replies our author: "knowledge amongst ourselves; knowledge of the actions and functions of the animal machine. *Its* influence will *soon* (?) be felt by the public." Something more than this sort of knowledge is wanted, we suspect. If there was more honest, upright principle abroad, more love of simple truth and of single-minded philanthropy, and less lust of money and of popular applause, more regard, in short, to those duties which M. Simon has with so much feeling and eloquence enforced in his "*Deontologie Medicale,*" the very fountain-source of quackery would be dried up. It is the heart, more than the head, that is generally at fault. Physiology can't put things right, in spite of all that Dr. Hall says in its praise: nay, there may be empiricism and charlatanerie in it as well as in other departments of science; witness the doings of more than one of the French school in the present day. Our author's mind is much more of a physiological or ætiological, than of a practical, cast. What the Germans call "naturforschung," appears to be the grand aim and end of his existence. His every thought seems to be taken up with striving to discover the *why* and the *wherefore* of vital actions. Truly his motto should be—

Felix qui potuit rerum cognoscere causas.

He tells us, in one passage, that "there is still ample scope for a useful treatise on the physiology of death and of dying. The great HUNTER has treated briefly of 'dissolution.' It would be interesting, and perhaps practically useful, to trace the *modes* in which each fatal disease of each organ destroys life." His restless inquisitive glance is thus peering about everywhere, and gathering food for the exercise of his ever-active mind. He makes discoveries, where other people only see simple recognised facts. For instance, every practical physician has been long well aware that the existence of dysphagia or dyspnœa, in cases of Hemiplegia, is always a most unfavourable omen. Dr. Hall is able to tell us the *why*:—the Spinal, as well as the Cerebral, system is involved. That this very love, however, of seeking to explain everything he meets with, sometimes leads him to take things supposed for things demonstrated, must strike every reader of his works: it is an almost inevitable consequence of his mental physiognomy. Thus, he lays it down as proved, that the muscular tissue of the intestines *is paralysed* in inflammation of their peritoneal coat; and then he goes on to say—"ought we to give purgative medicines, when the tissue, which they excite into contraction, is incapable of its function?" We admit the soundness of the practical precept, but not of the physiological interpretation.

There is certainly an ingenious subtlety in all his reflections; but it is often difficult to perceive their pertinence or value at the bed-side of the sick. The tenesmus and strangury in cases of Dysentery and Inflammation of the neck of the Bladder are declared to be 'reflex actions.' But what of that? Does such knowledge enable us the better to relieve those symptoms? Again; we are told that "the physiology of the nervous system is the very key to the act of parturition, and the very foundation of the Obstetric Art." What say Drs. Locock and Ramsbotham to this declaration? And then, with respect to the next assertion—that "in medical jurisprudence, physiology is everything"—we must leave it to Dr. Christison and other zealous labourers in that department of science to decide upon its truth. We, in our ignorance, had fancied that Chemistry had a good deal to do with the business.

Chap. III. consists of a mere scrap from a lecture on the subject of the *vis nervosa;* it contains nothing that need detain us. In the closing paragraph, "a knowledge of the true spinal system is" declared to be "to diseases of the Nervous System, in some degree, what the stethoscope is to diseases of the Thorax." This comparison, (which, by-the-bye, is repeated two or three times,) we fear, will hardly hold. Equally inapplicable is the reflection that, "what the heart is to the circulation of the blood, the spinal marrow is to the functions of the nervous system, the very source and centre of its operations." What then becomes of the cerebrum and its functions?

We are next presented with "A Succinct View of the Nervous System;" such as every reader of our author's writings must have met with a dozen of times. How edifying is the closing sentence:—

"I will conclude by the detail of an easy experiment, which any one may perform on the common frog:

1. Remove the brain;
2. Remove all the viscera.

The first operation removes the centre of the cerebral system; the second, almost every branch of the ganglionic.

"3. What then *remains? The spinal* MARROW; the true spinal *marrow,* hitherto undistinguished from the spinal *chord* of cerebral nerves, and the spinal *connections* of the ganglionic system." P. 21.

We have then a chapter on the "Influence of Emotion on the Health and in inducing and modifying Diseases." Chap. VII., on the same subject, may be briefly noticed along with it.

Dr. Hall is of opinion that the *seat* of the passions, and likewise of the sense of pain,* is in the *medulla oblongata;* for these powers or faculties remain when the *cerebrum* is reduced to its minimum. "The arm, when perfectly paralysed to volition in Hemiplegia, is agitated by emotion; and emotion exerts its influence on the heart, the intestinal tube, the secretions, &c."

---

* In a subsequent passage, it is said that pain "has its further seat, in many of the cases which come before the physician, in the *ganglionic* system. If this view of the subject be just, it follows that the trifacial nerve, so subject to be the seat of pain, contains ganglionic filaments, of which, indeed, from other considerations, there is little doubt."

The following extract, on the Location of the different Mental Faculties or endowments in the encephalic mass, gives a connected view of our author's opinions on a dark and difficult theme, and affords a good sample of his inquisitive character.

" The Intellect seems to be seated in the upper part of the cerebrum. In proportion as the development of this is defective, the being is unintellectual; he is an *idiot.*

" Its more manifest phenomena are connected with a certain set of nerves which may be termed Cerebral, and which are employed in conveying Sensations towards the central intellectual organ, and Volition from that organ.

" The Emotions are, I believe, seated lower down. The idiot, though deficient in intellect, is frequently swayed by the most violent emotions, and especially by the most extreme Fear, and the most violent Anger.

" The emotions display themselves principally, I believe, along that part of the nervous system which I have denominated the *true spinal,* or excito-motor.

" The Desires and the Passions are seated lower still. The idiot, who is so deprived of intellect as not to approach articles of food set before him even, swallows portions of that food placed within the mouth, with the most extreme avidity and voracity.

" The Instincts appear to me to be complicated in a high degree; and to combine—1, an act of volition; 2, the display of desire and passion; and 3, an act, probably unintellectual, and of an excito-motor character.

" The lark ascends the atmosphere by an act of volition, with the lively expression of a sexual passion, and probably keeps on the wing by an excito-motor act.

" What the lark does for its mate, the swallow does in search of food. It undertakes a long flight or aërial journey, urged by desire, guided by its intellect, sustained by the excito-motor principle—which is not subject to fatigue,—with the express object of the search of food.

" In the same manner, the bee provides its honey, and forms its honey-comb; this act combining mysteriously the influence of volition and of passion, whilst the actual deposit of its wax, and of its honey, may be effected, like those of deglutition and of inspiration—certainly not less wonderful or inscrutable,—by an excito-motor power impressed by the Creator for this purpose, as in other acts apparently of design.

" Thus also the beaver builds its hut, and the bird its nest,—impelled by passion, guided by volition, and aided, probably, by the excito-motor power." P. 32.

It is quite unnecessary to adduce any examples of the Influence of the Feelings on the Body in health and disease; as we treated of this interesting subject at considerable length in a recent number of this Journal.*

We have heard, within the last year or two, of Reflex Cerebral, as well as of Reflex Spinal, Actions. Dr. Hall does not admit the propriety of the language:

" There is an interesting fact which I cannot pass over: a disgusting object frequently acts on the spinal and ganglionic systems so as to induce sickness and faintness. This has been called a reflex cerebral action. It is mere emotion." P. 43.

Does one interpretation or mode of speech explain the matter better than the other?—we cannot tell.

The remarks on " Sleep," in Chap. VI., are not likely, we should think, to win the assent of many. Our author, ever anxious to find a reason for

---

* Vide Medico-Chirurgical Review for October 1845.

everything, suggests the following most singular explanation of the cause of this condition.

"I am of opinion, and I shall have to repeat the observation, that a state of contraction of certain muscles of the neck takes place, analogous to that of the orbicularis palpebræ, as sleep comes on; that certain veins are compressed; that congestion of the brain takes place; and, lastly, as a consequence of this last, *sleep*." P. 26.

A few pages farther on, we read :—

"Sleep, then, seems to be induced by any event which first gently excites and then gently lulls the attention. The influence of volition is removed. The levator palpebræ, a purely voluntary muscle (?), is consequently paralyzed; and the orbicularis—a muscle purely of excito-motor action (?)—is allowed its uncontrolled play; and the eye-lids close.

"Does any thing of this kind take place elsewhere? I have sometimes imagined that it does, and that it is *the* event which induces sleep. Certain muscles may be muscles principally of excito-motor action; and when volition is withdrawn from the other muscles of the neck, *they* may contract like the orbicularis, and gently compress the jugular veins, and so induce congestion of the brain, and sleep,—and, as we so often observe, attacks of apoplexy and of epilepsy. But I repeat that it is a mere conjecture—to be accepted for what it is worth, and a mere foil, if unfounded, to excite others to efforts more fortunate." P. 35.

That Sleep should be owing to congestion of the brain; that it should be "allied to Epilepsy;" that it should be apt to induce an attack of this disease, "by allowing the excito-motor power to act uncontrolled by volition;" and that "sleep and emotion induce similar *results,* acting on different principles," are so many positions which we gladly leave to our readers to discuss at their leisure, before inviting slumber to their eyes.

Chapters VIII. and IX. will not detain us. The first of these treats briefly of "Galvanism as a test of the Irritability of the Muscular Fibre." The second contains a few cursory remarks on those Paralytic Affections —often associated with spasmodic contractions in other parts—which occasionally occur during dentition.

"Are these effects the immediate effects of teething on the dental nerves? or are they induced through the medium of some morbid condition of the spinal marrow?

"This is a question which, I think, has never been investigated in the only way in which an accurate result can be obtained—by careful post-mortem examination. It is therefore all conjecture." P. 49.

We alluded, in our review of Dr. Hall's first volume, to his extravagant laudation of his own mode of scarifying the gums. He rebukes the whole profession for their want of faith in his advice :—

"To relieve the state of the alveolæ to which I have adverted, we must scarify the gums immediately contiguous to them; we must divide the very blood-vessels which feed them!—frequently—daily, or two or three times a day. But to this simple proposition objections still are made. Alas for our profession!" P. 50.

Chapter XI. is headed thus :—"The Dura Mater excitor; Diagnosis of Diseases of the Brain." It consists of a double report, one by Dr. Hall and the other by Mr. Smith, of an experiment which these *gentle*men performed on a dog, with the view of ascertaining, we are informed, "whether the Dura Mater, being supplied by a nerve usually excitor (the trifacial), is in itself excitor." We shall give our author's own account of this most humane and enlightened procedure; only adding that the poor tortured animal continued to breathe, until the medulla oblongata and upper part of the spinal marrow were removed!

"We removed a portion of the cranium of a spaniel dog, just over the *left* hemisphere of the cerebrum.

"We made pressure—gradually augmented: the dog became quiet, appeared to sleep; the eye-lids closed; the breathing became audible, stertorous, slow, sighing, the pupils contracted; at length, spasmodic twitchings were observed, and, afterwards, continued spasms: the eyes rocked, the tail was drawn, and then all the limbs were agitated and became stiffened.

"We removed the pressure, but the brain had been torn; the spasms ceased, the breathing became natural, the eye-lids opened, but the pupils remained contracted. The dog was *hemiplegic,* supporting his *right* side against the wall. We repeated this; the result was the same.

"We irritated the *nostril* and induced *sighing,* sometimes followed by sneezing, sometimes not. We irritated the meatus auditorius externus, and the head and limbs were much agitated. Sensibility was not entirely removed.

"We removed the cerebrum entirely: the pupils became permanently *dilated.* On irritating the motor oculi, the eye-lids were a little drawn by the corrugator supercilii, and closed and drawn towards the outer angle of the eye; but there was no action of the *levator.* The eyes rocked.

"On irritating the *dura mater* in various parts, these and other movements were also induced.

"On drawing the cerebellum forwards, convulsive movements were induced; but neither laceration of the cerebrum nor cerebellum seemed to induce muscular contraction.

"When the animal appeared *blind,* approaching the candle induced closure of the eye-lids; as did always touching the eye-lashes.

"The flow of saliva was constant, and in large quantity." P. 66.

Now this experiment, we are informed, has important bearings upon practical medicine. Let us see what these are. It illustrates, we are told, the effect of Irritation of the Dura Mater, and shews that a certain degree of pressure on the brain induces mere apoplexy, while a higher degree of that pressure induces convulsions and spasmodic affections. So valuable is the instruction derived from the experiment, that reference is made to it in no fewer than three of the subsequent chapters. In one of these, after favouring his readers with a mutilated report of a case of Puerperal Convulsions, the phenomena of which are said to have been precisely similar to those manifested by the poor mangled animal, Dr. Hall informs them (no one, certainly, could have discovered it for himself) that they must now "perceive the advantage of Experimental Physiology in its relation to Clinical medicine."!!

If such be the basis on which science is to be built, and such be the means to promote its advancement; if this be the sort of physiology that is set forth as "the only foundation for practical medicine," and this the KNOWLEDGE that is proclaimed as the sure corrective of quackery and the true exalter of our profession—it were better, we think, that science should be buried in the oblivion of Alchemy and Astrology, or be doomed to execration with that truculent Theology, which has impiously taught man to take the sword of persecution into his hand, and to pervert the glad tidings of mercy to mankind into the wild tocsin of cruelty and death, than that this revolting exhibition of animal pain and suffering should be, from year to year continued in our schools. And what, pray! are the real benefits that even the perpetrators of these atrocities must acknowledge themselves to have been thereby conferred on the art of healing?—for we suppose that there is scarcely any one who will justify them upon any other ground. Have we learned to treat any of the diseases of the nervous system more successfully, after all the horrible butcheries that have been committed within the course of the last five-and-thirty years, than did the wise and able men of last century, the Boerhaaves, the Cullens, or the Heberdens? Was Sydenham, may we ask, educated in the school of blood and torture? Was Mead a vivisector? Did Jenner's great thought originate in mutilating experiments upon animals? Did Pinel learn his lessons of philanthropic usefulness from witnessing the writhings of tortured life? Was Baillie's consummate skill in diagnosis derived from the dissection of living brutes?—or was Laennec's grand discovery the offspring of such a deed? Did Abercrombie offer up hecatombs of dogs and rabbits on the altar of science (!), ere he wrote his admirable work upon disease of the Brain? Has Andral ever been a fellow-labourer with Magendie or Longet? or has Louis himself—the beau-ideal of a physician in our author's opinion—reaped his laurels from that "knowledge of the state of the living morbid actions which," Dr. Hall declares, "must guide us in practice;" and not rather from that very pursuit which is somewhat slightingly called by him "crude and *post-mortem* morbid anatomy?"

We regret to say that the experiment, which has called forth these remarks, is far from being the only one related, or alluded to, in these "Practical Observations and Suggestions." At page 57, we read thus:—

"It is possible, by a peculiar mode of proceeding, to destroy or remove the spinal marrow, leaving the circulation of the blood subsistent. In this case, if the stomach, or intestine, or one of the limbs, be crushed, it seems to arrest the circulation entirely and at once; the injury acting, of course, through the medium of the ganglionic system only. Dr. Stilling has recently stated that, when the spinal marrow is entirely absent, no such effect is produced. He has not, I believe, deduced any inference from this experiment. The obvious inference would be, that the intra-spinal structure, and not the ganglia, is the centre also of reflected influence on the heart, and probably on all the internal organs. But the experiments require careful repetition" P. 58.

Is not the scientific *sang-froid* of the last sentence most edifying?

Chap. XIV. treats of "The Nature of Inflammation." One or two passages call for notice. The following description of the Capillaries is surely remarkable rather for its novelty than for its truth.

"These vessels have no distinct course,—no distinct successive unions, nor successive divisions,—no character of artery or vein, or of a tube of any kind. They pursue their course irregularly amongst the tissues,—sometimes uniting, sometimes separating,—uniting and dividing again,—so as to produce no appearance of a rectilinear, or indeed of a linear, tube. On the contrary, the course of these vessels is irregular, and varied in every way. It is such as one may imagine, or may have seen, to be given in water poured in moderate quantity over an uneven surface.

"Every thing, in a word, induces the belief that these capillary vessels are, in fact, not vessels, not tubes, but mere canals or channels formed amidst the tissues, like gutters in a chalky cliff. The mode of their continual junction and disjunction; the effect of certain re-agents on the capillary circulation (of a solution of common salt applied to the web of a frog, for example); seem to prove this peculiar character of the capillary vessels.

"Besides their rectilinear course, the veins and arteries are generally disposed *singly*, however deep the tissues. The capillary vessels, on the other hand, exist in great number, in whatever respect we consider them.

"From these and other considerations, I am of opinion that the term *blood-channels* would be a more just denomination for this part of the circulating system." P. 75.

It is somewhat curious that our author himself will not abide by his own description. In numerous passages of his volume, both before and after the extract now quoted, we read of the "(so called, but mis-named) capillary vessels," "capillary tubes," and never once of "blood-channels," as here proposed.

Here is Dr. Hall's theory of Inflammation in a few words:

"1. We have, first, adhesion of the blood-corpuscles in the blood-channels.
"2. This leads to Obstruction, and
"3. This to Congestion.
"4. We have then hypercrisis, or augmented *secretion;*
"5. Hypertrophy, or augmented size of the *old*, and the formation of *new*, vessels;
"6. Hyperendosmosis;
"7. Augmented *Absorption*, or Ulceration, Phagedæna, &c.
"8. Death, Sloughing, Gangrene." P. 90.

The various chapters, although occupying upwards of 100 pages, on "Puerperal Diseases, Puerperal Peritonitis, Puerperal Stomachal and Irritation, on the Effects of Loss of Blood in the Puerperal State, the Diagnosis of Puerperal Disease, the fatal effects of Blood-letting in Puerperal Affections, and the effects of previous Disorder on the Puerperal State," are most vexatiously overcharged with continual repetitions, and, after all, contain nothing but what has been perfectly well known to the public for the last twenty-five years at least. Almost all the recent treatises on the Diseases of Women exhibit a much more faithful and instructive picture of the phenomena of these morbid conditions, their diagnosis and treatment, than will be found in the present volume. Nay, we have no hesitation in saying, that it would be utterly impossible for any one to distinguish the truly inflammatory affections of the abdominal and pelvic viscera from those disorders, arising from intestinal irritation, fæculent accumulations, &c., which simulate them, if he trusted to the descriptions given of their distinguishing and characteristic phenomena by our author. The treatment, moreover, laid down by him is, in many respects, very far from being judicious. With respect to Dr. H.'s favourite dogma of making the induction of syncope a test as to the propriety of blood-letting, and the standard or measure as to the extent to which it may be carried, very few experienced men, we should think, can receive it as a rule of practice. Inflammatory affections of the abdominal or pelvic viscera in puerperal women will rarely admit of those large and ample blood-lettings, required for their subjugation under ordinary circumstances. To say, therefore, "that nothing should preclude of this remedy, but the actual existence of the state of sinking," is alike unsafe and most indiscreet. Is there not, too, a very misleading discrepancy of opinion upon an important practical point, in the following two passages, taken from different chapters in the present volume? At page 89, we read thus:

"It is well known that blood-letting is better borne in inflammation than in other diseases, and in inflammation of the serous membranes and the parenchymatous substance of organs, than in inflammation of the mucous membranes. This fact has become of great practical value. It is mentioned here only to complete the list of the phenomena of inflammation. The fact itself seems to *coincide* with the disposition to form the buffy coat, and with the augmented proportion of fibrine." P. 89.

Compare this with what we find at page 139.

"I have already noticed that one of the characteristics of intestinal irritation is the susceptibility to syncope upon blood-letting. This is, of course, much more remarkable upon a second or third blood-letting, than upon a first use of the lancet. I have now to add, that no dependence can be placed upon the appearance of the blood drawn. This may be much buffed and cupped, in the puerperal state, without the existence of inflammation; and in cases of the most decided inflammation, these appearances of the blood may be but little observed." P. 139.

What with there being in many instances of Puerperal Inflammation, according to our author, neither rigor, heat of surface, acceleration of the pulse, sickness, not even suppression of the lochial discharge, nor any buffy appearance of the blood when drawn, it must be a difficult thing indeed to distinguish the existence of the disease. But the experienced physician knows full well that there are other symptoms to guide his diagnosis, besides merely the circumstance of abdominal "pain, induced or aggravated upon pressure," which is declared by our author to be "pathognomonic of the disease."

Chap. XXVIII. is a reprint of our author's paper on the "Inverse Ratio which subsists between the Respiration and Irritability in the Animal Kingdom," that appeared in the Philosophical Transactions for 1832. Reference is actually made to the drawings which accompanied that paper, but which are altogether omitted in the present volume! Dr. Hall coolly informing his readers that he is *compelled* (by what?) to refer them to the Transactions for the drawing of the instrument which he describes. This is an easy off-hand sort of manner which great men only, we suppose, are permitted to assume.

This Chapter, as well as the following one on "Hybernation," contains many most ingenious and, what is better, sound views on physiology, which reflect the highest credit on our author's penetration and talent for research. The latter more especially is a beautiful specimen of a well-planned and most ably-conducted investigation of a scientific subject. It would alone serve to give Dr. Hall a high rank among physiological writers. As, however, the facts and conclusions of these papers are, or at least ought to be, quite known to the profession at large, being incorporated in various recent works on physiology, it would be altogether unsuitable to dwell upon them here.*

---

* A very ample analysis of Dr. Hall's Memoir on Hybernation will be found in the Medico-Chirurgical Review, for January 1840.

Chap. XXX. is a curious example of the restless impatience of Dr. Hall to have something to say upon every subject. It is entitled, " On the Influence of Air, Exercise, Bathing, Clothing." Regular moderate exercise is declared to be good; excessive exertion and fatigue must be injurious, inducing, through over-stimulus, a state of *fever*. So says Dr. H. Nay, he goes so far as to assert that the chief forms of acute and slow fevers, which are not specific, are principally owing to over-exertion and fatigue!

A little further on, we are told that " the rules for taking air, exercise, rest; for bathing and clothing; for food; for soil and climate; still remain to be physiologically deduced and fully unfolded!" In our ignorance, we had supposed that much had been done of late years in the investigation of these hygienic enquiries; alas! it all turns out to be a mistake. When will the spirit of " a profound physiology" be applied to these every-day matters? Take an example from our author on the subject of Dress.

> " The clothing should be such as to preserve the temperature, without oppressing the patient on one hand, or exposing him too much to changes in the atmospheric temperature on the other. In our variable climate, flannel should always be worn next the skin; the upper clothing should vary with the external temperature, the direction of the wind, the degree of damp, &c. Too much clothing in warm weather, and too little in severe, are equally injurious." P. 293.

We need scarcely say that so intelligent an observer as Dr. H, is a decided enemy to the present absurd fashion of exposing children's legs and arms to the sharp cold of winter, with the view of *hardening* them. Much mischief and "many heart-pangs" may often be traced to this foolish fashion. Tuberculous disease is a not unfrequent consequence:—" experiments on rabbits prove this!"

Not less characteristic of our author, is his 33rd Chapter, *On the fatal accident to the American diver.* The reader will perhaps remember that this unfortunate man accidentally hung himself upon Waterloo Bridge before a crowd of spectators, assembled to see him leap into the river. From the complete absence of struggling or other signs of suffering, our benevolent author believes that " the poor culprit suffers not a moment after suspension." In confirmation of this idea, he says:

> " I once saw a dog strangled by means of a cord, tied with extreme tightness round the neck. I expected to witness great struggles: there were none. There was not a movement until after some seconds, when convulsive efforts took place. A snake, suspended by the neck, ceases to move that instant." P. 304.

Really we were not aware that suffocation may possibly be so easy a death. Here is a proposal founded upon this idea:—

> " Might we not also avert the sufferings of that part of the brute creation which is slaughtered for our food and sustenance, and thus spare them the pang of the knife, and the pain of dying? Let (in?) sensibility be first induced, by a tightened cord, applied so as to compress the jugular and other veins of the neck, and then let the large vessels be divided as at present." 305.

No sooner is utterance given to this expression of humane feeling than we are again forced to enter the shambles of the operative physiologist, to seek for knowledge in the struggles of suffering life.

> " In the new edition of M. Flourens' admirable work, an account is given of an experiment, in which the cerebrum and cerebellum being removed, and the pneumogastrics divided, in a pigeon, the respiration still continued.

"Now it is well known that the respiration in birds, as in insects, is *diffused.* It is ascertained that the different segments of the insect possesses distinct nerves and nervous centres (analogues of the medulla oblongata) for respiration. This is not the case with birds; but,

"1. If the air cells throughout the body be, like those of the lungs, supplied with nerves;

"2. If these nerves be derived from the spinal nerves, like other nerves of the general frame; and,

"3. If these spinal nerves possess the *excitor* property of the spinal nerves in other animals;

"If these things be, then the result of the experiment is precisely what the theory of the reflex nature of the respiratory acts would have led us to anticipate.

"When, in the mammalia (especially the young), the cerebrum and cerebellum are removed, the respiration continues as a purely reflex action, excited principally through the pneumogastric nerves; when these are divided in addition, a few and rare acts of respiration occur, from the influence of the trifacial and spinal nerves. In birds, these spinal nerves are as really excitors of respiration as the pneumogastric itself; they are excitors of the normal respiration; under their sole influence, respiration may continue for hours." P. 307.

And straightway a new series of manglings and mutilations is announced as being highly desirable, and likely to produce important results !*

Chap. XXXVI., "On the plan of Observation of Diseases of the Nervous System," is a reprint of our author's fourth Memoir, read before the Medico-Chirurgical Society, and published in their Transactions: vide Vol. VI. New Series, and this Journal for January 1842.

As we noticed this paper very fully at the time of its first appearance, it is unnecessary to do more at present than merely mention its title. It is full of clever suggestions, many of which have a practical bearing; others are ingenious, but fanciful.

We must not, however, dismiss this chapter, without expressing our utter astonishment at finding an actual repetition, word for word, of a letter respecting Lady ———'s "most interesting case" of spasmodic tic, with which our author favoured the readers of the last volume of these "Observations and Suggestions." A good tale is said to be not the

---

* While this sheet was passing through the press, we stumbled upon the following admirable remarks in the newly-published volume of Dr. Latham: they contain, as will be seen, a gentle but severe rebuke of the operative physiology so highly lauded by our author.

"Disease is a great physiological teacher. Perhaps it is the greatest of all. It institutes experiments which we cannot imitate, and so tells us many things which, but for it, we should never know. I never laid bare a living brain, a living spinal marrow, or a living heart. I never took up a living nerve with the forceps, or noted the behaviour of those organs, severally or reciprocally, under modes of irritation which were of my own contrivance; yet, I have read of experiments which I never performed and never could bear to see; and I *may* have learned something from them; something, how dearly purchased!"—*Lectures on Clinical Medicine,* Vol. II., p. 17.

worse of being twice told; but the saying only holds true, we believe, of *vivâ voce* narration. Here, however, we have the benefit of it in clear legible print. Is not this too bad? There is an audacious trifling with the patience of the public in such an act, that fairly calls for rebuke and reprobation. But, in short, nothing connected with the name or doings of Dr. Marshall Hall but merits public attention. By the bye, he tells us that he had once the intention of withdrawing from practice and proceeding to Vienna to prosecute his investigations; "but that intention was thwarted." We sincerely trust, for the sake of his posthumous fame, that he will never think of an autobiographical sketch. Like an excentric ex-Chancellor of the day, no man could write down his reputation but himself.

The last of Dr. Hall's papers in this volume is entitled, "Plan proposed for the Institution of Blood-letting." We have already alluded to his views upon this subject, and expressed our partial dissent from them; but, as the subject is one immediately connected with practice, it may deserve a somewhat ampler notice. Here is the position, then the illustration, and lastly its practical application.

"Different diseases induce in the constitution different powers or susceptibilities in regard to the effects of loss of blood. Each disease appears, indeed, to possess its own peculiar and intrinsic virtue in this respect. This is determined by placing the patient perfectly erect, and bleeding to incipient syncope: the quantity of blood which flows is the measure of the protective influence of the disease in one class of cases, and of its influence in superinducing a susceptibility to the effects of loss of blood in the other.

"An interesting scale of diseases may be formed representing these properties. It would begin with congestion of the head, or tendency to apoplexy; inflammation of the serous membranes, and of the parenchymatous substance of various organs would follow; then acute anasarca; and lastly, inflammation of the mucous membranes. This part of the scale would be divided from the next by the condition of the system in health. Below this would be arranged fever, the effects of intestinal irritation, some cases of delirium, reaction from loss of blood, and disorders of the same class with hysteria, dyspepsia, chlorosis, and cholera morbus." P. 351.

"The practical application of this fact consists chiefly in its affording a rule for blood-letting in all cases in which this measure is required to be fully instituted; a guard against undue blood-letting, both in this and some other cases; and a source of diagnosis.

"The quantity of blood which flows, when a patient requiring full blood-letting is placed upright and bled to deliquium, seems accurately proportionate to the exigencies of the case. In inflammation, much blood should be taken; and much blood will flow before deliquium is induced: in irritation, little blood should be drawn; and there is early syncope from blood-letting. The quantities are even accurately suited, not only to the exigencies of the disease, but to the powers of the system; at least, so it appears to me, from considerable experience." P. 353.

Dr. H. informs us that persons in health, and of moderate strength, will generally faint, if bled in the erect posture, on taking 15 ozs. of blood; that in Brochitis little more is borne to be lost than in health: that a stout person in fever (what form of fever is not stated) will frequently faint on losing 10, 12 or 14 ounces. He has known a patient, of good strength affected with Cholera, to faint on taking 4 ozs., although she had shortly before borne to lose 20 ozs. without faintness, under the influence of inflamed mamma:—there is certainly nothing wonderful in that. He then endeavours to explain the cause of the different degrees of the *tolerance* of blood-letting in different diseases, thus:—

"In all those cases in which the circulation of the heart and larger arteries alone is affected, and especially in such as involve irritation, or exhaustion, there is early syncope on taking blood. But in such cases as consist in an affection of the capillary circulation, and especially such of these as affect the head, it requires the abstraction of much blood to induce deliquium. Syncope is prevented by the influence exerted by this state of the capillary circulation over that of the heart and larger arteries, and over the whole system, and especially over the circulation within the brain; and it does not entirely subdue the morbid action of the capillary vessels even when induced. To induce syncope in pure fever, we have then but to subdue the state of reaction in the heart and larger arteries. In inflammation, we have not only to do this, but to overcome the influence of a permanent morbid action of the capillaries: this is especially observed in inflammation of the serous membranes, and within the head." P. 353.

But surely Apoplexy, the disease of all others in which loss of blood is said to be best borne, cannot properly be called an "inflammation within the head." There must therefore be some other element besides that of mere inflammatory action to be taken into account, in determining the tolerance of blood-letting in different states of the system. Have not the insensibility and unconsciousness of the individual a good deal to do with the amount of blood which he can lose without fainting? It is not easy to account in any other way for the enormous depletions that can be borne in cases of cerebral congestion, whether this be of spontaneous or of traumatic origin. In sleep, too, the amount of blood, that may be lost without syncope, is decidedly much greater than in the waking state. In short, whenever mental or emotional influence is suspended, the tendency to fainting is very remarkably diminished. Dr. Hall seems to leave, almost entirely, out of consideration the agency of the mind in increasing or diminishing the liability to syncope in different cases. We are therefore by no means disposed to agree with him in regard to the practical value of his *formula*, as a means of diagnosis in doubtful cases, laid down in these words:—"If much blood has flowed before syncope occurred, we must suspect inflammation; if little, we must suspect that, however similar the symptoms, the case is in fact of a different nature—perhaps irritation, perhaps exhaustion."

Again, when there is sharp pain present, more especially when the seat of the pain is not internal or visceral, a patient will generally bear a much more copious blood-letting than he probably could an hour before. We often witness an illustration of this, in the attempts to induce faintishness in cases of recent dislocation. In acute Rheumatism, too, the sharp articular suffering may have a good deal to do with the tolerance of blood-letting, which is so conspicuous a symptom in this disease. The same remark holds good of almost all acute spasmodic or convulsive diseases; witness Tetanus.

With respect to the proposal of our author that "in every case, in which full blood-letting is to be instituted, the patient should be placed perfectly erect, and bled to the very first appearance of deliquium," we need scarcely say that cases are continually occurring in practice, where it is advisable to have a larger amount of blood drawn than may be just necessary to induce faintishness in the upright position. Was such a rule invariably followed, the remedy would often fail of producing the permanent effects upon the system that are desired.

Dr. Hall seems to be quite aware himself of the inapplicability of his rule for blood-letting in certain cases of disease :—

"There are two exceptions to the rule which I have proposed, which I would briefly mention. In some cases of fever requiring blood-letting, the patient cannot support the erect position: in such a case, the arm should be first prepared, and then the patient should be gently raised and supported in the upright position, carefully avoiding all muscular effort; the vein should then be promptly opened. On the other hand, in the case of congestion of the brain from exhaustion, there is not such early syncope from blood-letting as might be expected; and yet it is obvious that the system cannot bear the loss of blood. I have known this to obtain in exhaustion from undue lactation." P. 358.

We should like to know what cases of Fever these are to which Dr. H. refers, and in which the patient, although he cannot support the erect position, should be gently raised up into it, and then be bled to approaching deliquium. Is the loss of blood, may we ask, ever advisable under such circumstances? The case clearly supposes the existence of great debility; and surely general blood-letting, and that too carried to faintishness, is not the remedy that a judicious physician would employ to relieve either pain, congestion, or even inflammatory action under such circumstances. The local depletion of blood by the cupping-glasses or by leeches will very generally be preferred. The other case alluded to, viz. that of Congestion of the Brain from undue lactation or other exhausting disorders, serves but to confirm the remarks which we have made above, relative to the influence of torpor or unconsciousness as an important element to be taken into consideration in any attempt to investigate the different degrees of tolerance of blood-letting in different states of the system. But without pursuing this subject, who, may we enquire, would ever dream of *general* blood-letting in the case supposed? And now we must bid adieu to our author.

No one can reasonably deny that he is a man of uncommon powers of mind, of unwearied industry, and indomitable energy; that he has long and successfully laboured in the vineyard of medical science; that his varied writings proclaim him to be a keen observer and a subtle reasoner; that his discoveries in physiology have justly gained for him a foremost place among the investigators of that science; that to him alone belongs the rare honour of having first clearly propounded and manfully worked out one of the most perplexing problems in the history of animal life, unravelling the meshes of a most intricate perplexity, and pointing out the clue, the Ariadne thread of guidance, to all future explorers through the labyrinth of former darkness and confusion; that he has thus not only stamped the impress of his thoughts on the medical literature of the day (one of the surest proofs of original genius), and achieved for himself a world-reputation, but that he has also added to the intellectual and scientific fame of his country; which may now—thanks to the labours of Harvey, Bell and Hall—justly claim the undisputed glory of being the birth-place of the two mightiest discoveries in physiological science. But these are not the only merits of our author. His more practical writings, even from the very outset of his professional career, have contributed, in no trifling degree, to render the symptomatology and discrimination of many diseases more accurate and intelligible; and, although he has been apt on some occasions to exaggerate the importance of a few phenomena or signs as guides for diagnosis and treatment, we cordially award him the praise of having done good service to the practice of Medicine.

How comes it then that the works of such a man should often have called forth so much censure and disapprobation? That Dr. Hall has met with most unhandsome and unjust treatment from several of our contemporaries, cannot now be gainsayed by any impartial witness. While one party has vainly attempted to rob him of his fair fame, another has, with equal impotence, thought to quench the torch of discovery by refusing to act as one of its honoured light-bearers. No such reproaches can be made by any one against us. We have uniformly maintained the justice of his claims as a great and original investigator of physiological science against all envy and detraction. Ten years ago, we spoke of his labours in these words:—"He has evolved a simple fact (that of involuntary muscular contractions following the irritation of the corresponding sensory nerves, as long as the part retains its connection with the spinal cord) into an extensive and ingenious theory, applied it to the solution of complex phenomena previously surrounded with obscurity, and has done a great deal towards clearing the way to a *precise* acquaintance with the Functions of the Nervous System. Such appears to us to be the meed, which even a parsimonious critic must award to Dr. Hall."* In October 1841, we gave what is perhaps the most complete analysis of his researches and discoveries in neurological science, to be found anywhere out of his own writings; an analysis that has been more than once referred to in works on the diseases of the cerebro-spinal system. The ample and enlarged notices too, which we bestowed on the four memoirs of Dr. Hall, published in the Medico-Chirurgical Transactions, must have rendered our readers thoroughly and minutely acquainted with the many interesting facts which he has adduced, and with those doctrines by which he has so ingeniously sought to weave the whole into a tissue of the most admirable construction.†

It cannot, therefore, be fairly said that we, at least, have shewn any unwillingness to recognise the great merits of our author in various departments of medical literature; and therefore it was that we felt the less hesitation in withholding our approbation from the first volume of these "Practical Observations and Suggestions." Would that we could consci-

---

* Medico-Chirurgical Review, No. 52, p. 306.

† *Vide* Medico-Chirurgical Review, Nos. 63, 67, and 71. We gladly avail ourselves of this opportunity to direct the attention of our readers to the eloquent and ably-argued summary of Dr. Hall's neurological discoveries and doctrines that recently appeared in successive numbers of the Lancet, for August 8th, 15th, and 29th. Some will probably say that the papers are a little too eulogistic; but no one can fail to admire the lucid exposition, vigorous reasoning, and graceful diction which they display.

entiously make up for the censure of the last by the praise of the present one! But this cannot be; and the fault lies solely and altogether with Dr. Hall himself. He is one of those characters that *will not* correct his errors: for most assuredly he is not so blind as not to see them, or so indifferent to applause as not to peruse what is written of him. Pertinacity is the very woof of his character. He deems it a point of honour never to change or concede aught, except to himself. Jealous of his own, sometimes to very paltriness, he is continually obtruding it upon the public; if it won't attend to him to-day, he is determined to force it to do so to-morrow. Hence his continued repetition, *usque ad nauseam*, of the same facts, dogmas, or doctrines, varied somewhat it may be in some accessory points, but not unfrequently dressed out in the very same attire. Thus it is, as we have seen, that he has actually republished in the present volume the very same letter which made its appearance in the former series of these Observations, and which, if we mistake not, had already seen the light elsewhere before. This unworthy blemish of his writings has been pointed out more than once; but, as a matter of course, no respect is ever paid to the remarks of others. Dr. Hall has not only erred, but he has suffered, much from this arrogancy of character. Had he but learned to listen more to the voice of friendly counsel, and often less to the suggestions of his own impetuous mind; had he been more conciliatory in his manners, more charitable and forbearing in his disposition; had he attached less importance to (his panacea) *knowledge*, and more to *wisdom;* had he trusted more for the advancement of his opinions to the irresistible (although slow and silent) force of simple truth, and been less disposed to thrust them upon others by the ruder weapons of controversy and dogmatic iteration, he would have avoided many of those strifes, heartburnings, and detractions which have beset his path, and might have more peacefully enjoyed the sweets of that reputation which he has so meritoriously achieved. Whatsoever be the spirit in which these remarks may be received by him, it matters little to us. Our duty is simple, and straight before us;—cordially to award praise where praise is justly due, and frankly to express our censure of error, whether this be in the shape of ignorance, wilful negligence, or vain presumption.

